# Horizontal and Vertical Integration of Health Care Providers: A Framework for Understanding Various Provider Organizational Structures

**DOI:** 10.5334/ijic.4635

**Published:** 2020-01-20

**Authors:** Jessica Heeringa, Anne Mutti, Michael F. Furukawa, Amanda Lechner, Kristin A. Maurer, Eugene Rich

**Affiliations:** 1 Mathematica Policy Research, US; 2 Agency for Healthcare Research and Quality, US

**Keywords:** vertical integration, horizontal integration, health systems, integrated care

## Abstract

**Introduction:**

Current U.S. policy and payment initiatives aim to encourage health care provider accountability for population health and higher value care, resulting in efforts to integrate providers along the continuum. Providers work together through diverse organizational structures, yet evidence is limited regarding how to best organize the delivery system to achieve higher value care.

**Methods:**

In 2016, we conducted a narrative review of 10 years of literature to identify definitional components of key organizational structures in the United States. A clear accounting of common organizational structures is foundational for understanding the system attributes that are associated with higher value care.

**Results:**

We distinguish between structures characterized by the horizontal integration of providers delivering similar services and the vertical integration of providers fulfilling different functions along the care continuum. We characterize these structures in terms of their origins, included providers and services, care management functions, and governance.

**Conclusions and discussion:**

Increasingly, U.S. policymakers seek to promote provider integration and coordination. Emerging evidence suggests that organizational structures, composition, and other characteristics influence cost and quality performance. Given current efforts to reform the U.S. delivery system, future research should seek to systematically examine the role of organizational structure in cost and quality outcomes.

## Introduction

The U.S. health care delivery system is characterized by complexity. Individuals obtain health insurance through private or public sources that target certain patient populations on the basis of employment, age, disability status, income status, military service, or other factors, while a portion of society remains uninsured for various reasons. The result of this heterogeneous coverage environment is that multiple players, including federal and state policymakers and private payers, influence the health care delivery system and patient care. These players develop eligibility policies, determine which services will be covered, contract with provider networks, establish provider reimbursement models, and engage in some level of care management. At the same time, hospitals, physicians, and other providers, such as post-acute care providers, are structurally and contractually organized in diverse arrangements, with varying levels of autonomy.

Compared to many other industrialized countries, the U.S. health care system has been found to incur higher costs but yield lower access, quality, and population health outcomes [1]. Like other countries, U.S. policymakers and payers have viewed integrated care as a strategy for improving health care quality and efficiency. “Integrated care” has been defined as “a coherent set of methods and models on the funding, administrative, organizational, service delivery and clinical levels designed to create connectivity, alignment and collaboration within and between the cure and care sectors” [2].

Various U.S. federal and state policies, as well as local market factors, have influenced how, and which types of, providers work together to care for patient populations. Although the integration of providers has been a persistent strategy, the type of integration and its goals have varied over time. Between the 1980s and mid-1990s, the U.S. health care delivery system experienced a shift in the predominant type of integration—from horizontal integration, when organizations acquire or integrate with other organizations that provide the same or similar services such as multihospital systems or multispecialty practice organizations [3]—to vertical integration, when organizations acquire or integrate with organizations offering different levels of care, services, or functions such as hospital ownership of physician practices [3,4].

Major policy shifts, such as the Patient Protection and Affordable Care Act (ACA) of 2010, have in part fueled a resurgence in efforts to promote integrated care. Increasingly, hospitals, physicians, and other providers are consolidated into health systems [5,6]. This trend toward the vertical integration of various provider types has occurred while there has been a shift in U.S. policymaker attention to improving health outcomes and patient-centeredness as elements of health care value [4]. The ACA established multiple programs and policies to test new delivery system and payment models that emphasize improved access to care and care management along the care continuum, furthering the incentives for integration among providers [7]. For example, growing financing and delivery system models, such as accountable care organizations (ACOs) and patient-centered medical homes (PCMHs), are built on a foundation of primary care, shared accountability, and improved care management [4,7].

Following the ACA, private and public sector payers have been shifting from fee-for-service reimbursement models to risk-based models that encourage shared accountability for the total costs of care between payers and providers. Collectively, these emerging payment and delivery system models encourage coordination and integration across providers to ultimately improve quality and cost outcomes [8].

Despite the adoption of integration as a primary reform strategy, there is presently a gap in evidence regarding which underlying structural changes in local health care delivery systems are most effective in achieving higher value care [9]. To address this gap in evidence, the U.S. Agency for Healthcare Research and Quality (AHRQ) established the Comparative Health System Performance Initiative [10]. As a formative step in this initiative, we conducted a review of the literature spanning 10 years to identify the core elements of organizational structures in the U.S. health care system and describe them with respect to their included health care providers and services, care management functions, and administrative oversight of included providers. A clear accounting of these common organizational structures is foundational to ongoing work to understand the core characteristics of systems that are associated with improved quality and cost outcomes.

## Methods

In September 2016, we conducted a narrative review of 10 years of literature to identify definitional components, care management functions, and administrative oversight of key organizational structures. To characterize care management, we looked for information regarding each organizational structure’s role in coordinating and managing the care of defined patient populations and available resources and capacity within each structure to facilitate care management, such as health information technology. To understand how the administrative oversight of included providers varied across organizational structures, we examined descriptions of constructs such as the governance of included providers, the nature of relationships among providers (for example, contractual relationships), and the extent to which included providers retain professional autonomy in each structure.

For this narrative overview, we began with a foundational set of articles and then used an iterative search strategy to capture additional relevant literature [11]. Specifically, we started with a set of 22 prominent articles describing a range of organizational approaches to health care delivery systems. To select these articles, we solicited key references from experts in the field associated with the AHRQ Comparative Health System Performance Initiative, with the goal of including historical and contemporary literature addressing a range of health care delivery organizational strategies in the aggregate. This initial list included seminal reviews, taxonomies of health care systems, and original studies of various provider organizational structures (see Appendix A for the list of 22 articles). We then used a “snowballing” approach to identify other relevant literature. Specifically, we searched the Scopus database of peer-reviewed and grey literature to identify additional articles building from the reference lists of these 22 articles, dating back to 2007. We then searched Google Scholar to search for new articles that cited the original 22 articles. We supplemented these searches with additional key author searches and targeted hand searching to fill in gaps on identified organizational structures for health care delivery systems.

After removing duplicates, three reviewers screened the titles and abstracts of 1,750 articles for relevance. Articles were included if they: (1) addressed the U.S. health care delivery system and (2) focused on characterizing health care provider organizations and/or health systems. In total, we analyzed 87 publications for this review. The full list of reviewed articles is presented in Appendix B. We used NVivo 11, a software that supports qualitative analysis, to analyze included texts. Three researchers developed, tested, and refined a code list to apply to the literature text. The team coded a shared set of five articles to ensure coding consistency, then independently coded the remaining pieces.

## Results

The literature identifies a wide variety of U.S. organizational structures and approaches to integrating care across providers. We group these structures in terms of horizontal and vertical integration and describe how they vary in terms of their goals, included providers, and key features. Table 1 provides a summary of each structure’s key features. We focus on care management and administrative oversight, which may influence the nature and magnitude of integration among included providers.

**Table 1:** Key features of the horizontally and vertically integrated structures.

Organization type	Included health care providers and services	Care management functions	Administrative oversight of providers

Horizontally integrated structures			

Single specialty group practice	Physicians Physician services	Varies depending on specialty represented	Hospitals, health plans, physicians, and other firms may own or manage single specialty practices, which could influence the degree of administrative oversight over included providers.
Multispecialty group practice	Physicians of various specialties Services vary depending on included specialties	May facilitate patient referral, improve care coordination, and be better-positioned to manage the costs of care [12,15]	Multispecialty group practices share governance and infrastructure, which can result in tighter management control; however, control can vary depending on factors such as size and whether the practice is physician-owned, owns a hospital, or is owned by hospital/system [12,15].
Independent practice association	Physicians Services vary depending on included specialties	Largely serve contracting role and provide administrative and contractual functions [15] May provide infrastructure services to support performance improvement and care management [20,32] May provide processes and resources to support care management such as disease registries, nurse care managers, etc. [14]	Physicians maintain independent ownership and management of practices, while the independent practice association primarily negotiates contracts with health plans [16,40].
Virtual physician networks	Physicians Services vary depending on included specialties	Entities that organize these networks, such as medical foundations or state Medicaid agencies, may provide care coordination networks; certain infrastructure resources, such as health information technology and information exchange; and care management services to member physicians, who in turn could use those services in the provision of care [15,18,20]	These networks tend to be characterized by less formal bureaucratic control [15].
Multihospital systems	Two or more hospitals Primarily hospital services, which may include inpatient and ambulatory services	Varies depending on included service; in vertically integrated models, multihospital systems may have care functions that a more analogous to integrated delivery systems.	As multihospital systems are characterized by shared ownership or management, administration may have more direct control over included hospitals, including care processes, shared organizational missions, and the like. However, they may also maintain separate hospital boards and executives, despite shared asset ownership [24].

**Vertically integrated structures**			

Physician hospital organization	Hospitals and their affiliated physicians Hospitals and physician services, which vary depending on included specialties	Facilitate managed care contracting, provide administrative services to physicians, facilitate natural referral relationships around one hospital, and manage ambulatory care facilities where physicians work [15,28] Closed physician-hospital organizations selectively contract with physicians on the basis of quality and cost performance and have exclusive relationships with physicians and close relationships with hospitals, which may facilitate care coordination [28] May provide processes and resources to support care management [14]	Physicians maintain independent ownership and management of practices, while practices contract with health plans through the organization [18,28].
Management services organization	Physicians and hospitals if hospitals are the owners of the organization	These organizations provide administrative and infrastructure services, which may include care coordination, care management services and health information technology, to physician members [12,27,28]	Management services organizations provide services to member physicians and contract with payers on behalf of member providers; however, providers largely retain independence.
Clinically integrated network	Primarily include physicians but may also include hospitals and other providers such as post-acute care providers. Services vary depending on network composition.	Providers seeking to form these networks must demonstrate integration clinically through a number of activities, including implementation of a program to evaluate and modify practice patterns and creation of a high degree of interdependence and cooperation among network physicians to control costs and ensure quality. Example features of programs include: Implementing systems to ensure appropriate utilization of services Deploying evidence-based practice standards and protocols Performance evaluation and feedback to included providers Case management and care coordination [29,30]	Providers are either integrated via ownership or contractual relationships; the clinical integration framework requires physicians to use consistent care protocols and to monitor quality, suggesting greater oversight and management of included providers [29,30].
Foundation model	Varies; primarily limited to physicians; however, in some states with corporate practice of medicine laws, certain hospitals such as nonprofit health corporations or federally qualified health centers, may employ physicians, provided physician autonomy is maintained [33]. Physician services are explicitly included, but these structures are often formed to facilitate collaboration between hospitals and physicians [28,33].	Varies; may have functions similar to integrated delivery systems in models where the physician organization and hospital have mutually exclusive contracting relationships [31]	A key feature of this model is the salaried employment of physicians by a non-profit entity; while employment may suggest greater management control of included providers, states with corporate practice of medicine laws – where these models are relevant – are explicitly focused on maintaining the clinical independence of physicians [31,33].
Integrated delivery system	Varies; may include hospitals, physicians, and other health care providers such as post-acute care providers, behavioral health, community-based organizations, as well as health plans [15,31,39] Comprehensive, full continuum of care [15,39]	Care coordination and information sharing along care continuum [13,15,31] Population health and care management [13] Data collection, analysis, and reporting capabilities to inform quality improvement [13,15,31] Health information technology capacity [15] Use of evidence-based practices [31] Interdisciplinary, team-based care [31]	Providers join systems through ownership or formalized contractual agreements, which typically establish some degree of administrative control. Administrative control may vary depending on the extent to which the system centralizes management activities, engages in physician-system integration, and employs physicians [12,28].

### Horizontally integrated organizational structures

**Single specialty group practices.** Historically, U.S. physicians practiced as individual providers in “solo” practice. Thus, the simplest form of horizontal integration is the single specialty group practice. These organizations can be of varying sizes and are composed of physicians with a common specialty, although in the modern era of sub-specialization, related specialties may be aggregated into one organization. For example, non-invasive cardiologists, interventional cardiologists, and electrophysiologists work together in a single specialty cardiology group. Physicians primarily form single specialty practices to achieve economies of scale and gain market share, while they may also be seeking professional management or infrastructure investments [12,13]. These practices may also be owned by hospitals, health plans, or other firms [12].

**Independent practice associations.** Independent practice associations (IPAs) are loosely, contractually integrated networks of independent physicians and physician groups that are primarily organized to engage in risk-based contracting with payers [12,14]. IPAs initially emerged in response to the growth of managed care in the United States during the 1990s but continue to be a relevant model in the context of payment reform, as they help network physicians assume and share financial risk while also enabling them to maintain their independence [14,15,16]. These organizational structures may also provide infrastructure services and create processes for quality improvement and care management [12]. As an example, Hill Physicians Medical Group, one of the largest IPAs in the United States, contracts with health insurers on behalf of its large primary care and specialist physician network [17]. Shortell, Casalino, and Fisher (2010) observed that many IPAs have evolved over time “into more-organized networks of practices that are actively engaged in practice redesign, quality improvement initiatives, and implementation of electronic health records” [18, p. 1295].

**Multispecialty group practices.** Multispecialty group practices (MSGPs) bring together a diverse group of physicians, including primary and specialty care physicians, “who share common governance, infrastructure, and finances, and refer patients to one another for services offered within the group” [13, p. 2]. Physicians tend to form these organizations to share governance, resources, and patients and essentially achieve greater care coordination [13,15]. Shortell, Casalino, and Fisher (2010) noted, “Because they include multiple specialties, they can provide most care that patients need within the group…” [15, p. 54]. Because of their scope, MSGPs have sometimes been described as having “highly developed mechanisms for providing coordinated clinical care” [18, p. 1294]. Further, MSGPs, such as the Mayo Clinic, may have strong affiliations and referral relationships with a specific local hospital, which may transition into formal vertical integration relationships [15,19].

**Virtual physician networks.** Virtual physician networks are less formalized, regional networks intended to provide infrastructure, care management, care coordination networks, and other resources to providers to support the provision of integrated, organized care locally [15,18,20]. Often formed to serve rural areas or otherwise underserved U.S. patient populations, they may be payer- or provider-driven and are often facilitated by individual providers, state Medicaid agencies, medical foundations, or similar organizations [18,20]. Such networks can serve as the basis for more substantive integration strategies such as Medicaid ACOs. For example, Minnesota’s Integrated Health Partnership’s ACO model includes a virtual model enabling providers not affiliated with a hospital or IDS to form virtual networks for the purposes of serving as a Medicaid ACO [21].

**Multihospital systems.** Multihospital systems are characterized by “horizontal integration of facilities…that provided similar acute care services in multiple locations.” [22, p. 15]. The University of Pennsylvania Health System, composed of three hospitals, is an example [23]. Burns and Pauly (2002) noted that these systems “feature common asset ownership but separate system versus hospital boards and executives” [24, p. 131]. These systems emerged to help hospitals achieve economies of scale and improve access through an expanded delivery network through integration of hospitals in the late 1980s to mid-1990s [4,24]. However, many evolved into vertically integrated structures through acquisition of physician practices, ambulatory centers, and post-acute care providers, among other entities [4,15]. Thus, to the extent these systems also include other care providers, they would be more appropriately classified as a form of vertical integration.

### Vertically integrated organizational structures

**Physician-hospital organizations.** Physician-hospital organizations (PHOs), such as Advocate Health System in Chicago, are a form of physician-hospital integration, albeit a looser one than certain other models such as medical foundations [14,15,24]. PHOs entail a formal partnership between hospitals and all or some of their affiliated physicians for the purposes of contracting with one or more health plans [12,13,15,16]. Physicians and hospitals form PHOs to achieve greater alignment while maintaining autonomy and being governed separately [14,15,24]. Indeed, Shortell and colleagues (2014) defined a PHO as an “organisational form that is less formally integrated into a system, but is based on alignment across clinicians and hospitals.” [25, p. 23] Wise and colleagues (2012) noted that PHOs generally have some form of affiliation agreement that allows physicians and the hospital(s) to work cooperatively while being governed independently [26]. Physicians in these arrangements may share care management and information technology resources with other practices [14].

**Management services organizations.** Management services organizations (MSO) are entities owned by a hospital or physician-hospital joint venture that purchase physical assets of participating physicians and provide administrative services to physicians for a fee [24,26]. Often grouped with other forms of physician-hospital integration, MSOs may entail exclusive contracting relationships between hospitals and physicians [14,27]. Providers formed these organizations for the purposes of contracting with health plans and to obtain administrative and infrastructure support [12,28]. Because MSOs provide a range of administrative and infrastructure support services to member physicians, they may play a role in supporting the provision of certain care management functions.

**Clinically integrated networks.** Clinically integrated networks (CINs) are composed of physicians, hospitals, and potentially other providers who would otherwise be competitors but come together in a joint venture that meets the clinical integration criteria specified by the U.S. Department of Justice and Federal Trade Commission [29]. These models emerged in in response to antitrust regulations that require provider organizations that would be competitors to share substantial financial risk or be clinically integrated [29,30]. Accordingly, they must have an ongoing quality improvement program underway and certain shared functionalities in place such as clinical protocols, case management, physician performance monitoring and feedback, and clinical information and health information technology [29,30].

**Foundation models.** Foundation models are corporate entities, usually nonprofit entities, that employ or engage in professional services agreements with physicians and exist within nonprofit hospital systems [31,32]. These models are a form of physician-hospital integration that emerged in specific U.S. states with corporate practice of medicine laws prohibiting corporations from practicing medicine [28,31,33]. These models often are similar to some definitions of IDSs in that they are intended to achieve close physician-hospital integration and are often characterized by exclusive contracting relationships between the foundation model practice and the affiliated hospital [28,31]. For example, Kaiser Permanente, often described as an IDS model [15] is actually three distinct units: Kaiser Foundation Health Plan, Kaiser Foundation Hospitals, and Permanente Medical Groups that are contractually connected [23]. Because they are intended to facilitate hospital-physician integration, these models may provide a range of comprehensive health care services and care functions.

**Integrated delivery systems.** The literature is replete with discussions of IDSs, without clear consensus on the definition [34]. Many definitions of IDSs focus on the structural aspects of the entity (e.g., formal, legal relationships among organizations), while others emphasize delivery system functions such as providing a comprehensive continuum of care and accepting accountability for patient population or community health. For example, Enthoven (2009) described an IDS as “an organized, coordinated, and collaborative network that links various healthcare providers to provide a coordinated, vertical continuum of services to a particular patient population or community.” [35, p. S284] Similarly, Shortell and McCurdy (2009) noted that an IDS may include “various alliances and partnerships formalized through contractual relationships.” [36, p. 370] Other definitions of IDSs are more specific that the entities composing the IDS will have common ownership. Recognizing these definitions, Casalino (2014) noted that “the lowest common denominator structural definition of an IDS would be that it is an organization that includes one or more hospitals, plus medical groups, within a single ownership structure.” [37, p. 1880] While their specific organizational structures can vary, IDSs tend to be characterized by comprehensive health care services, shared accountability for the cost and clinical outcomes of defined patient populations, and a focus on improved integration and coordination among health care service providers, that may include post-acute care providers, behavioral health, and community-based organizations [13,35,38,39].

## Discussion

We identified a range of horizontally and vertically integrated organizational structures in the United States and described how they vary in terms of their origins, included providers and services, care management functions, and governance. We also describe how different models relate to various local market pressures, payment policies, and provider regulations in the United States.

Current trends in U.S. health care emphasize patient-centered models of care that call for better integration and coordination of health, and sometimes social services, to meet patient needs [4,12,34]. This shift in part relates to the emerging focus on chronic disease management and population health, which require engagement of diverse providers along the care continuum. In a review of over 25 years of international literature on health systems integration, Evans et al. (2013) identified major shifts in integration strategies, including a shift from horizontal to vertical integration strategies and a shift from acute- or institution-centered models to those emphasizing greater coordination among community-based health and social services [4]. Although policy strategies emphasize integration, U.S. policymakers currently lack clear evidence on the forms of integration that are most effective. This challenge has motivated the AHRQ Comparative Health System Performance Initiative [10].

For example, in a systematic review of literature examining vertical integration, Machta et al. (2018) found that vertical integration in the U.S. was associated with higher performance on some measures of quality (often measured for patient populations with specific conditions), but not for measures of cost or resource utilization, while evidence on the influence of vertical integration on patient-centered outcomes was lacking [41]. In another review of the literature on integrated delivery networks, Goldsmith et al. (2014) found little evidence that hospital-physician structural integration alone yields improved cost and quality outcomes, pointing to research suggesting that organizational change should be coupled with enabling care management and governance processes to support performance improvement [39,42]. A 2013 review of integrated care strategies by Hwang et al. examined literature that spanned organizational arrangements—from MSGPs to IDSs, finding that integrated care was associated with improved quality outcomes. However, the authors noted the role of organizational factors such as use of electronic health records or implementation of quality improvement initiatives that could influence outcomes [43].

In view of this quandary, some private and public payment policies are promoting the growth of ACOs wherein diverse networks of providers become collectively accountable for the care of defined patient populations [15]. These models of care are characterized by provider collaborations among hospitals, primary and specialty care physicians, behavioral health, community-based organizations, and potentially other providers along the care continuum. By the end of 2017, over 10 percent of the U.S. population received care through such an ACO [44]. Some researchers have observed that ACOs attempt to achieve many of the same goals of vertically integrated delivery systems without requiring formal structural integration [45], and emerging evidence suggests that the model can yield desired outcomes under certain circumstances.

For example, in a systematic review of ACO outcomes, Kaufman et al. (2017) found mixed evidence on the effects of ACOs on utilization, processes of care, and patient outcomes, with the most consistent outcomes being reduced inpatient and emergency department use and improved adult preventive care and chronic disease management measures [46]. While the ACO model has been associated with some improved outcomes, performance varies among ACOs. Research on ACOs suggests that organizational factors, such as the types of providers who are participating in the model, may partially explain this variation. Specifically, McWilliams and colleagues (2018) compared reductions in spending between physician-group ACOs and hospital-integrated ACOs from 2012 to 2014 in Medicare, the public health insurance program primarily serving older Americans. The authors found that physician-group ACOs, on average, accrued increasing cost savings during the study period, while hospital-integrated ACOs did not yield cost savings on average during the same period [47]. Other research suggests the characteristics of participating providers may influence ACO performance. Lewis and colleagues (2018) found that providers participating in ACOs may have different levels of experience with care management and care coordination—core tenets of the ACO model—which could influence performance [48]. Furthermore, many ACOs are composed of primarily independent providers, which may create challenges for standing up the infrastructure and governance needed to achieve the high integration of care management intended for ACOs [48]. Taken together, these findings suggest that myriad factors, including organizational structure and composition, may influence the degree to which providers participating in ACOs are integrated and achieve high performance.

Finally, our review pointed to the various efforts to integrate providers, but how these models influence the patient experience is less clear. Observers have cautioned that formal provider integration may not result in more integrated care from the patient perspective. For example, Kerrissey and colleagues (2017) conducted a national survey of Medicare beneficiaries to examine the association between medical group practice structural integration and patient experiences of care integration, finding an inconsistent association between provider structural characteristics and patients’ perceived integration of care [49]. Thus, additional work is needed to understand how provider organizational structures influence the patient experience.

## Conclusion

We provided a framework for understanding the array of U.S. provider organizational structures that integrate local providers to promote higher value care. We describe the diverse relationships between health care providers that have arisen in the U.S. context as providers respond to policy and payment reforms, in order to aid researchers and policymakers seeking to characterize the further evolution of health care delivery. Given current efforts to reform the U.S. delivery system, future research should seek to systematically examine the role of organizational structure in cost, quality, and patient-centered outcomes. Such work will be essential for developing a better understanding of the structures that are most effective in helping providers achieve higher value care for their communities.

## Additional Files

The additional files for this article can be found as follows:

10.5334/ijic.4635.s1 Appendix A. List of 22 initial articles that formed the basis of the search strategy.

10.5334/ijic.4635.s2 Appendix B. Full List of Reviewed Literature.
