# Supplementary material for: Horizontal and Vertical Integration of Health Care Providers: A Framework for Understanding Various Provider Organizational Structures
Source: Int J Integr Care. 2020 Jan 20;20(1):2. doi: 10.5334/ijic.4635 (PMC6978994; doi:10.5334/ijic.4635)
Supplement: Appendix A. — List of 22 initial articles that formed the basis of the search strategy. [file ijic-20-1-4635-s1.pdf]

Appendix A: List of 22 initial articles that formed the basis of the search strategy

Bazzoli GJ, Shortell SM, Ciliberto F, Kralovec PD, Dubbs NL. Tracking the changing provider landscape: Implications for health policy and practice. Health Aff (Milwood), 2001 Nov; 20(6): 188-196.

Bazzoli GJ, Shortell SM, Dubbs N, Chan C, Kralovec P. A taxonomy of health networks and systems: Bringing order out of chaos. Health Serv Res, 1999 Feb; 33(6): 1683-1717.

Bazzoli GJ, Shortell SM, Dubbs NL, Luke RD. Rejoinder to taxonomy of health networks and systems: A reassessment. Health Serv Res, 2006 Jun; 41(3 Part 1): 629-639.

Burns LR, Goldsmith JC, Sen A. Horizontal and Vertical Integration of Physicians: A Tale of Two Tails. In: Goes J, Savage GT, Friedman L. editors. Annual Review of Healthcare Management: Revisiting the Evolution of Health Systems Organizations. Advances in Health Care

Chukmaitov A, Harless DW, Bazzoli GJ, Carretta HJ, Siangphoe U. Delivery System Characteristics and Their Association with Quality and Costs Care: Implications for Accountable Care Organizations. Health Care Manage Rev, 2015 Apr-Jun; 40(2): 92–103

Cutler D and FS Morton. Hospitals, Market Share, and Consolidation. Journal of American Medical Association, 2013 Nov; 310(18): 1964-1970.

Devers KJ, Shortell SM, Gillies RR, Anderson DA, Mitchell JB, Erickson KLM. Implementing Organized Delivery Systems: An Integration Scorecard. Health Care Manage Rev, 1994 Summer; 19(3): 7–20.

Dubbs NL, Bazzoli GJ, Shortell SM, Kralovec PD. Reexamining organizational configurations: An update, validation, and expansion of the taxonomy of health networks and systems. Health Serv Res, 2004Feb; 39(1): 207-220.

Enthoven AC. Integrated Delivery Systems: The Cure for Fragmentation. Am J Manag Care, 2009 Dec; 15(10 Suppl): S284–S290.

Epstein AM. Promoting delivery system integration to foster higher value care slow progress ahead. JAMA Intern Med, 2013 Aug; 173(15): 1456-1457.

Kreindler SA, Larson BK, Wu FM, et al. Interpretations of Integration in Early Accountable Care Organizations. Milbank Q, 2012; 90(3): 457–483.

Luke RD. Taxonomy of health networks and systems: A reassessment. Health Serv Res, 2006 Jun; 1(3 Part 1): 618-628.

Maeda JL, Lee KM, Horberg M. Comparative health systems research among Kaiser Permanente and other integrated delivery systems: A systematic literature review. Perm J, 2014 Summer; 18(3): 66-77.

McWilliams JM, Chernew ME, Dalton JB, Landon BE. Outpatient care patterns and organizational accountability in Medicare. JAMA Intern Med, 2014 Jun; 174(6): 938-945.

Piña IL, Cohen PD, Larson DB, Marion LN, Sills MR, Solberg LI, Zerzan J. A Framework for Describing Health Care Delivery Organizations and Systems. Am J Public Health, 2015 Apr; 105(04): 670-679.

Robinson JC, Casalino LP. Vertical Integration and Organizational Networks in Health Care. Health Aff (Milwood). 1996; 15(1): 7–22.

Shih A, Davis K, Schoenbaum S, Gauthier A, Nuzum R, McCarthy D. Organizing the U.S. Health Care Delivery System for High Performance. New York: The Commonwealth Fund, 2008 Aug. Publication No. 1155. Available from: https://www.commonwealthfund.org/sites/default/files/documents/___media_files_publications_fund_report_2008_aug_organizing_the_u_s__health_care_delivery_system_for_high_performance_shih_organizingushltcaredeliverysys_1155_pdf.pdf.

Shortell SM, Bazzoli GJ, Dubbs NL, Kralovec P. Classifying health networks and systems: Managerial and policy implications. Health Care Manage Rev, 2000 Fall; 25(4): 9-17.

Shortell SM, Wu FM, Lewis VA, Colla CH, Fisher ES. A taxonomy of accountable care organizations for policy and practice. Health Serv Res, 2014 Dec, 49(6): 1883-1899.

Singer SJ, Burgers J, Friedberg M, Rosenthal MB, Leape L, Schneider E. Defining and Measuring Integrated Patient Care: Promoting the Next Frontier in Health Care Delivery. Med Care Res Rev, 2011 Feb; 68(1): 112-27.

Valentijn PP, Boesveld IC, van der Klauw DM, et al. Towards a taxonomy for integrated care: A mixed-methods study. Int J Integr Care, 2015 Jan-Mar; 15: e003.

Wu FM, Shortell SM, Lewis VA, Colla CH, Fisher ES. Assessing differences between early and later adopters of accountable care organizations using taxonomic analysis. Health Serv Res, 2016 Dec; 51(6): 2138-2329.
