# Supplementary material for: Horizontal and Vertical Integration of Health Care Providers: A Framework for Understanding Various Provider Organizational Structures
Source: Int J Integr Care. 2020 Jan 20;20(1):2. doi: 10.5334/ijic.4635 (PMC6978994; doi:10.5334/ijic.4635)
Supplement: Appendix B. — Full List of Reviewed Literature. [file ijic-20-1-4635-s2.pdf]

Appendix B: Full List of Reviewed Literature

Alidina S, Rosenthal M. Coordination within Medical Neighborhoods: Insights from the Early Experiences of Colorado Patient-Centered Medical Homes. Health Care Manage Rev, 2016 Apr-Jun; 41(2): 101-112.

Amelung VE. Provider-Based Managed Care Organisations and Products. In: Amelung, VE editor. Healthcare Management: Managed Care Organisations and Instruments. Heidelberg, Germany: Springer; 2013. p. 63-81.

American Medical Association. Corporate practice of medicine. Chicago, IL: American Medical Association, 2015. [Cited 2017 May 22]. Available from: https://www.ama-assn.org/sites/default/files/media-browser/premium/arc/corporate-practice-of-medicine-issue-brief_1.pdf.

Armitage GD, Suter E, Oelke ND, Adair CE. Health systems integration: state of the evidence. Int J Integr Care, 2009 Apr-June; 9(17): e82.

Bazzoli GJ, Shortell SM, Ciliberto F, Kralovec PD, Dubbs NL. Tracking the changing provider landscape: Implications for health policy and practice. Health Aff (Milwood), 2001 Nov; 20(6): 188-196.

Bazzoli GJ, Shortell SM, Dubbs N, Chan C, Kralovec P. A taxonomy of health networks and systems: Bringing order out of chaos. Health Serv Res, 1999 Feb; 33(6): 1683-1717.

Bazzoli GJ, Shortell SM, Dubbs NL, Luke RD. Rejoinder to taxonomy of health networks and systems: A reassessment. Health Serv Res, 2006 Jun; 41(3 Part 1): 629-639.

Billings J, de Weger E. Contracting for Integrated Health and Social Care: A Critical Review of Four Models. J Integ Care, 2015; 23(3): 153–175.

Bishop TF, Shortell SM, Ramsay PP, Copeland KR, Casalino LP. Trends in hospital ownership of physician practices and the effect on processes to improve quality. Amer J Manag Care, 2016 Mar; 22(3): 172-6.

Budetti PP, Shortell SM, Waters TM, et al. Physician and Health System Integration. Health Aff (Milwood), 2002 Jan; 21(1): 203-210.

Burns LR, Goldsmith JC, Sen A. Horizontal and Vertical Integration of Physicians: A Tale of Two Tails. In: Goes J, Savage GT, Friedman L. editors. Annual Review of Healthcare Management: Revisiting the Evolution of Health Systems Organizations. Advances in Health Care Management: Volume 15. West Yorkshire: Emerald Group Publishing; 2013. p. 39-117.

Burns LR, Muller RW. Hospital-Physician Collaboration: Landscape of Economic Integration and Impact on Clinical Integration. Milbank Q, 2008 Sep; 86(3): 375–434.

Burns LR, Pauly MV. Accountable Care Organizations May have Difficulty Avoiding the Failures of Integrated Delivery Networks of the 1990s. Health Aff (Milwood), 2012 Nov; 31(11): 2407-2416.

Burns LR, Pauly MV. Integrated Delivery Networks: A Detour on the Road to Integrated Health Care? Health Aff (Milwood), 2002 Jul; 21(4): 128-143.

Burns LR, Shah RJ, Sloan FA, Powell AC. The Impact of Hospital Ownership Conversions: Review of the Literature and Results from a Comparative Field Study. In: Savage GT, Fottler MD. editors. Biennial Review of Health Care Management: Meso Perspective. Advances in Health Care Management: Volume 8. West Yorkshire, England: Emerald Group Publishing; 2009. p. 171-229.

Burns LR, Wholey DR, McCullough JS, Kralovec P, Muller R. The Changing Configuration of Hospital Systems: Centralization, Federalization, or Fragmentation? In: Friedman LH, Savage GT, Goes J. editors. Annual Review of Health Care Management: Strategy and Policy Perspectives on Reforming Hospital Systems. West Yorkshire, England: Emerald Group Publishing; 2012. p. 189-232.

Carluzzo KL, Larson BK, Van Citters AD, et al. Monarch HealthCare: Leveraging Experience in Population Health Management to Attain Accountable Care. New York: The Commonwealth Fund, 2012 Jan. Publication No. 1573. [Cited 2018 Nov 26]. Available from: https://www.commonwealthfund.org/sites/default/files/documents/___media_files_publications_case_study_2012_jan_1573_carluzzo_monarch_case_study_01_17_2012.pdf.

Casalino LP. Categorizing Accountable Care Organizations: Moving Toward Patient-Centered Outcomes Research That Compares Health Care Delivery Systems. Health Serv Res, 2014 Dec; 49(6): 1875-1882.

Casalino LP. The Federal Trade Commission, Clinical Integration, and the Organization of Physician Practice. J Health Polit Policy Law, 2006 Jun; 31(3):569-85.

Casalino LP, Chen MA, Staub CT, Press MJ, Mendelsohn JL, Lynch JT, Miranda Y. Large Independent Primary Care Medical Groups. Ann Fam Med, 2016 Jan; 14(1): 16-25.

Casalino LP, Erb N, Joshi MS, Shortell SM. Accountable Care Organizations and Population Health Organizations. J Health Polit Policy Law, 2015 Aug; 40(4): 821-37.

Casalino L, Robinson JC. Alternative Models of Hospital-Physician Affiliation as the United Moves Away from Tight Managed Care. Milbank Q, 2008; 81(2): 331-351.

Casalino LP, Wu FM, Ryan AM, et al. Independent practice associations and physician-hospital organizations can improve care management for smaller practices. Health Aff (Milwood). 2013, 32(8): 1376-1382.

Christianson J, Carlin CS, Warrick LH. The Dynamics of Community Health Care Consolidation: Acquisition of Physician Practices. Milbank Q, 2014 Sep; 92(3): 542-567.

Chukmaitov A, Harless DW, Bazzoli GJ, Carretta HJ, Siangphoe U. Delivery System Characteristics and Their Association with Quality and Costs Care: Implications for Accountable Care Organizations. Health Care Manage Rev, 2015 Apr-Jun; 40(2): 92–103.

Colla CH, Lewis VA, Shortell SM, Fisher ES. First National Survey of ACOs Finds That Physicians Are Playing Strong Leadership and Ownership Roles. Health Aff (Milwood), 2014 Jun; 33(6): 964–971.

Cowen M, Halasyamani LK, McMurtie D, Hoffman D, Polley T, Alexander JA. Organizational Structure for Addressing the Attributes of the Ideal Healthcare Delivery System. J Healthc Manag, 2008 Nov-Dec; 53(6): 407-418.

Crosson FJ. 21st-Century Health Care - the Case for Integrated Delivery Systems. N Engl J Med, 2009 Oct; 1(361): 1324-1325.

Cuellar AE, Gertler PJ. Strategic Integration of Hospitals and Physicians. J Health Econ, 2006 Jan; 25(1): 1–28.

Cutler DM, Scott Morton F. Hospitals, market share, and consolidation. JAMA, 2013 Nov, 310(18), 1964-1970.

Devers KJ, Shortell SM, Gillies RR, Anderson DA, Mitchell JB, Erickson KLM. Implementing Organized Delivery Systems: An Integration Scorecard. Health Care Manage Rev, 1994 Summer; 19(3): 7–20.

Diana ML, Walker DM, Mora AM, Zhang Y. Vertical Integration Strategies in Healthcare Organizations. J Health Adm Educ, 2015 Spring; 32(2): 223-244.

Dubbs NL, Bazzoli GJ, Shortell SM, Kralovec PD. Reexamining organizational configurations: An update, validation, and expansion of the taxonomy of health networks and systems. Health Serv Res, 2004Feb; 39(1): 207-220.

Enthoven AC. Integrated Delivery Systems: The Cure for Fragmentation. Am J Manag Care, 2009 Dec; 15(10 Suppl): S284–S290.

Enthoven A. Curing Fragmentation with Integrated Delivery Systems: What They Do, What Has Blocked Them, Why We Need Them, and How to Get There From Here. In: Elhauge E. editor. The Fragmentation of U.S. Health Care. New York: Oxford University Press; 2010. p. 61-85.

Enthoven AC. What Is an Integrated Health Care Financing and Delivery System (IDS)? And What Must Would-Be IDS Accomplish to Become Competitive with Them? Health Econ Outcome Res Open Access, 2016; 2(2): 115-124.

Epstein AM. Promoting delivery system integration to foster higher value care slow progress ahead. JAMA Intern Med, 2013 Aug; 173(15): 1456-1457.

Epstein AM, Jha AK, Orav EJ, et al. Analysis of Early Accountable Care Organizations Defines Patient, Structural, Cost, and Quality-of-Care Characteristics. Health Aff (Milwood), 2014 Jan; 33(1): 95–102.

Essential Hospitals Institute. Integrated Health Care: Literature Review. Washington, D.C.: Essential Hospitals Institute, May 2013. [Cited 2018 Nov 26]. Available from: http://essentialhospitals.org/wp-content/uploads/2013/12/Integrated-Health-Care-Literature-Review-Webpost-8-22-13-CB.pdf.

Evans JM, Baker GR, Berta WB, Barnsley J. The Evolution of Integrated Health Care Strategies. In: Goes J, Savage GT, Friedman L. editors. Annual Review of Healthcare Management: Revisiting the Evolution of Health Care Systems. Advances in Health Care Management: Volume 15. West Yorkshire: Emerald Group Publishing; 2013. p. 125-61.

Fennell ML, Adams CM. US Health-Care Organizations: Complexity, Turbulence, and Multilevel Change. Annu Rev Sociol. 2011 Apr; (37): 205-219.

Fisher ES, Shortell SM, Kreindler SA, Van Citters AD, Larson BK. A Framework for Evaluating the Formation, Implementation, and Performance of Accountable Care Organizations. Health Aff (Milwood), 2012 Nov; 31(11): 2369-2378.

Fisher ES, Staiger DO, Bynum JPW, Gottlieb DJ. Creating Accountable Care Organizations: The Extended Hospital Medical Staff. Health Aff (Milbank), 2007; 26(1): w44–w57.

Fottler M., Savage GT, Blair JD. The future of integrated delivery systems: a consumer perspective. In: Wolper LF. editor. Health Care Administration: Managing Organized Delivery Systems. 5th ed. Sudbury, MA: Jones and Bartlett Publishers; 2000. p. 67-106

Gillies RR, Shortell SM, Anderson DA, Mitchell JB, Morgan KL. Conceptualizing and Measuring Integration: Findings from the Health Systems Integration Study. Hosp Health Serv Adm, 1993 Winter; 38(4):467–89.

Gitterman DP, Weiner BJ, Domino ME, McKethan AN, Enthoven AC. The Rise and Fall of a Kaiser Permanente Expansion Region. Milbank Q, 2003 Dec; 81(4): 567-601.

Goldsmith J, Burns LR, Sen A, Goldsmith T. Integrated Delivery Networks: In Search of Benefits and Market Effects. Washington, DC: National Academy of Social Insurance, 2015. [Cited 2018 Nov 26]. Available from: https://www.nasi.org/sites/default/files/research/Integrated_Delivery_Networks_In_Search_of_Benefits_and_Market_Effects.pdf.

Hwang W, Chang J, LaClair M, Paz H. Effects of Integrated Delivery System on Cost and Quality. Amer J Manag Care, 2013 May; 19(5): e175–e184.

Kodner D. All Together Now: A Conceptual Exploration of Integrated Care. Healthc Q, 2009 Oct; 13 (Spec): 6–15.

Kreindler SA, Larson BK, Wu FM, et al. Interpretations of Integration in Early Accountable Care Organizations, Milbank Q. 2012; 90(3): 457–483.

Kroch E, Champion RW, Devore SD, Kugel MR, Lloyd DA, Rothney-Kozlak L. Measuring Progress Toward Accountable Care. New York: The Commonwealth Fund, 2012. 32 p. Publication No.1652. Available from: https://www.commonwealthfund.org/sites/default/files/documents/___media_files_publications_fund_report_2012_dec_1652_kroch_measuring_progress_web_1212.pdf.

Landry AY, Erwin CO. Organization of Care. In: Jonas and Kovner's Health Care Delivery in the United States. New York: Springer Publishing Company; 2015. p. 183-211.

Larson BK, Van Citters AD, Kreindler SA, et al. Insights From Transformations Under Way at Four Brookings-Dartmouth Accountable Care Organization Pilot Sites. Health Aff (Milwood), 2012 Nov; 31(11): 2395-2406.

Laugesen MJ, France G. Integration: the firm and the health care sector. Health Econ Policy Law, 2014 Jul; 9(3): 295-312.

Luke RD. Taxonomy of health networks and systems: A reassessment. Health Serv Res, 2006 Jun; 1(3 Part 1): 618-628.

Maeda JL, Lee KM, Horberg M. Comparative health systems research among Kaiser Permanente and other integrated delivery systems: A systematic literature review. Perm J, 2014 Summer; 18(3): 66-77.

McCarthy D. Integrated Healthcare Delivery Models in an Era of Reform. In: Shiver JM, Cantiello, J. editors. Managing Integrated Health Systems. Burlington, MA: Jones and Bartlett, 2015. p. 1-24.

McCarthy D, Mueller K. Organizing for Higher Performance: Case Studies of Organized Delivery Systems—Series Overview, Findings, and Methods. New York: The Commonwealth Fund, 2009 Jul. 38 p. Publication No. 1288. Available from: https://www.commonwealthfund.org/sites/default/files/documents/___media_files_publications_case_study_2009_jul_1288_mccarthy_overview_report_final.pdf.

McWilliams JM, Chernew ME, Dalton JB, Landon BE. Outpatient care patterns and organizational accountability in Medicare. JAMA Intern Med, 2014 Jun; 174(6): 938-945.

Mostashari F. The Paradox of Size: How Small, Independent Practices Can Thrive in Value-Based Care. Ann Fam Med, 2016 Jan; 14(10): 5-7.

Muhlestein D, Gardner P, Merrill T, Petersen M, and Tu T. A Taxonomy of Accountable Care Organizations: Different Approaches to Achieve the Triple Aim. Washington, D.C.: Leavitt Partners, June 2014, 1-12.

Nolte E, McKee M. Integration and Chronic Care: A Review. In: Nolte E, McKee M. editors. Caring for People with Chronic Conditions: A Health System Perspective. Berkshire, England: McGraw Hill Open University Press; 2008. p. 64-91.

Page S. 'Virtual' Health Care Organizations and the Challenges of Improving Quality. Health Care Manage Rev, 2003 Jan-Mar; 28(1): 79-92.

Piña IL, Cohen PD, Larson DB, Marion LN, Sills MR, Solberg LI, Zerzan J. A Framework for Describing Health Care Delivery Organizations and Systems. Am J Public Health, 2015 Apr; 105(04): 670-679.

Robinson JC, Casalino LP. Vertical Integration and Organizational Networks in Health Care. Health Aff (Milwood). 1996; 15(1): 7–22.

Shields MC, Patel PH, Manning M, Sacks L. A Model for Integrating Independent Physicians into Accountable Care Organizations. Health Aff (Milwood), 2011 Jan; 30(1): 161–172.

Shih A, Davis K, Schoenbaum S, Gauthier A, Nuzum R, McCarthy D. Organizing the U.S. Health Care Delivery System for High Performance. New York: The Commonwealth Fund, 2008 Aug. Publication No. 1155. Available from: https://www.commonwealthfund.org/sites/default/files/documents/___media_files_publications_fund_report_2008_aug_organizing_the_u_s__health_care_delivery_system_for_high_performance_shih_organizingushltcaredeliverysys_1155_pdf.pdf.

Shortell SM. Commentary On Horizontal And Vertical Integration Of Physicians: A Tale Of Two Tails By Lawton Robert Burns, Jeff C. Goldsmith, and Aditi Sen. In: Goes J, Savage GT, Friedman L. editors. Annual Review of Health Care Management: Revisiting the Evolution of Health Systems Organization Advances in Health Care Management, Volume 15. West Yorkshire, England: Emerald Group Publishing; 2013. p. 119-124.

Shortell SM. Applying Organization Theory to Understanding the Adoption and Implementation of Accountable Care Organizations: Commentary. Med Care Res Rev, 2016 Dec; 73(6): 694-702.

Shortell SM, Bazzoli GJ, Dubbs NL, Kralovec P. Classifying health networks and systems: Managerial and policy implications. Health Care Manage Rev, 2000 Fall; 25(4): 9-17.

Shortell SM, Addicott R. A New Lens on Organizational Innovations in Health Care: Forms and Functions. In: Ferlie E., Montgomery K, Reff Pedersen A. editors. The Oxford Handbook of Health Care Management. Oxford: Oxford University Press; 2016. p. 45-71.

Shortell SM, Addicott R, Walsh N, Ham C. Accountable care organisations in the United States and England: Testing, evaluating and learning what works. London: The King's Fund, 2014. [Cited 2018 Nov 26]. Available from: https://www.kingsfund.org.uk/sites/default/files/field/field_publication_file/accountable-care-organisations-united-states-england-shortell-mar14.pdf.

Shortell, SM, Casalino LP, Fisher ES. How the Center for Medicare and Medicaid Innovation Should Test Accountable Care Organizations. Health Aff (Milwood), 2010 Jul; 29(7): 1293–1298.

Shortell SM, Casalino LP, Fisher ES. Achieving the Vision—Structural Change. In: Crosson FJ, Tollen LA. editors. Partners in Health: How Physicians and Hospitals Can Be Accountable Together. San Francisco: Jossey-Bass; 2010. p. 46-71.

Shortell SM, Colla CH, Lewis VA, Fisher E, Kessell E, Ramsay P. Accountable Care Organizations: The National Landscape. J Health Polit Policy Law, 2015 Aug; 40(4): 647-668.

Shortell SM, McClellan SR, Ramsay PP, Casalino LP, Ryan AM, Copeland KR. Physician Practice Participation in Accountable Care Organizations: The Emergence of the Unicorn. Health Serv Res, 2014 Oct; 49(5): 1519-1536.

Shortell SM, McCurdy RK. Integrated Health Systems. Information Knowledge Systems Management, 2009; 8: 369–382.

Shortell SM, Wu FM, Lewis VA, Colla CH, Fisher ES. A taxonomy of accountable care organizations for policy and practice. Health Serv Res, 2014 Dec, 49(6): 1883-1899.

Singer SJ, Burgers J, Friedberg M, Rosenthal MB, Leape L, Schneider E. Defining and Measuring Integrated Patient Care: Promoting the Next Frontier in Health Care Delivery. Med Care Res Rev, 2011 Feb; 68(1): 112-27.

Strandberg-Larsen M, Schiotz ML, Silver JD, et al. Is the Kaiser Permanente Model Superior in Terms of Clinical Integration?: A Comparative Study of Kaiser Permanente, Northern California and the Danish. BMC Health Serv Res, 2010 Apr; 10(91): 1-13.

U.S. Department of Justice and the Federal Trade Commission. Statements of Antitrust Enforcement Policy in Health Care. Washington, D.C.: U.S. Department of Justice and the Federal Trade Commission, 1996 Aug.

Valentijn PP, Boesveld IC, van der Klauw DM, et al. Towards a taxonomy for integrated care: A mixed-methods study. Int J Integr Care, 2015 Jan-Mar; 15: e003.

Valentijn PP, Schepman SM, Opheij W, Bruijnzeels MA. Understanding Integrated Care: A Comprehensive Conceptual Framework Based on the Integrative Functions of Primary Care. Int J Integr Care, 2013 Mar; 22; 13:e010.

Van Citters AD, Larson BK, Carluzzo KL, et al. Four Health Care Organizations' Efforts to Improve Patient Care and Reduce Costs. New York: The Commonwealth Fund, 2012 Jan. Publication No. 1571. Available from: https://www.commonwealthfund.org/sites/default/files/documents/___media_files_publications_case_study_2012_jan_1571_van_citters_dartmouth_aco_synthesis_01_12_2012.pdf.

Wise CG, Alexander JA, Green LA, Cohen GR. Physician Organization-Practice Team Integration for the Advancement of Patient-Centered Care. J Ambul Care Manage, 2012 Oct-Dec; 35(4):311-22.

Wu FM, Shortell SM, Lewis VA, Colla CH, Fisher ES. Assessing differences between early and later adopters of accountable care organizations using taxonomic analysis. Health Serv Res, 2016 Dec; 51(6): 2138-2329.

Yeager VA, Zhang Y, Diana ML. Analyzing Determinants of Hospitals' Accountable Care Organizations Participation: A Resource Dependency Theory Perspective. Med Care Res Rev, 2015 Dec; 72(6): 687-706.
